# Human Dectin-1 deficiency impairs macrophage-mediated defense against phaeohyphomycosis

**DOI:** 10.1172/JCI159348

**Published:** 2022-11-15

**Authors:** Rebecca A. Drummond, Jigar V. Desai, Amy P. Hsu, Vasileios Oikonomou, Donald C. Vinh, Joshua A. Acklin, Michael S. Abers, Magdalena A. Walkiewicz, Sarah L. Anzick, Muthulekha Swamydas, Simon Vautier, Mukil Natarajan, Andrew J. Oler, Daisuke Yamanaka, Katrin D. Mayer-Barber, Yoichiro Iwakura, David Bianchi, Brian Driscoll, Ken Hauck, Ahnika Kline, Nicholas S.P. Viall, Christa S. Zerbe, Elise M.N. Ferré, Monica M. Schmitt, Tom DiMaggio, Stefania Pittaluga, John A. Butman, Adrian M. Zelazny, Yvonne R. Shea, Cesar A. Arias, Cameron Ashbaugh, Maryam Mahmood, Zelalem Temesgen, Alexander G. Theofiles, Masayuki Nigo, Varsha Moudgal, Karen C. Bloch, Sean G. Kelly, M. Suzanne Whitworth, Ganesh Rao, Cindy J. Whitener, Neema Mafi, Juan Gea-Banacloche, Lawrence C. Kenyon, William R. Miller, Katia Boggian, Andrea Gilbert, Matthew Sincock, Alexandra F. Freeman, John E. Bennett, Rodrigo Hasbun, Constantinos M. Mikelis, Kyung J. Kwon-Chung, Yasmine Belkaid, Gordon D. Brown, Jean K. Lim, Douglas B. Kuhns, Steven M. Holland, Michail S. Lionakis

**Affiliations:** 1 Fungal Pathogenesis Section and; 2 Immunopathogenesis Section, Laboratory of Clinical Immunology and Microbiology (LCIM), National Institute of Allergy and Infectious Diseases (NIAID), NIH, Bethesda, Maryland, USA.; 3 Division of Infectious Diseases, McGill University Health Centre (MUHC), and Infectious Disease Susceptibility Program, Research Institute-MUHC, Montreal, Quebec, Canada.; 4 Department of Microbiology, Icahn School of Medicine at Mount Sinai, New York, New York, USA.; 5 Division of Intramural Research, NIAID, NIH, Bethesda, Maryland, USA.; 6 Research Technologies Branches, NIAID, NIH, Hamilton, Montana, USA.; 7 Bioinformatics and Computational Biosciences Branch, Office of Cyber Infrastructure and Computational Biology, NIAID, NIH, Bethesda, Maryland, USA.; 8 Laboratory for Immunopharmacology of Microbial Products, School of Pharmacy, Tokyo University of Pharmacy and Life Sciences, Tokyo, Japan.; 9 Inflammation and Innate Immunity Unit, LCIM, NIAID, NIH, Bethesda, Maryland, USA.; 10 Research Institute for Biomedical Sciences, Tokyo University of Science, Chiba, Japan.; 11 National Institute of Deafness and Other Communication Disorders (NIDCD), NIH, Bethesda, Maryland, USA.; 12 Laboratory of Pathology, Center for Cancer Research, National Cancer Institute (NCI), Bethesda, Maryland, USA.; 13 Radiology and Imaging Sciences and; 14 Department of Laboratory Medicine, NIH Clinical Center, NIH, Bethesda, Maryland, USA.; 15 Division of Infectious Diseases, Houston Methodist Hospital, Houston, Texas, USA.; 16 Center for Infectious Research, Houston Methodist Research Institute, Houston, Texas, USA.; 17 Division of Infectious Diseases, UCSF, San Francisco, California, USA.; 18 Division of Infectious Diseases, Mayo Clinic, Rochester, Minnesota, USA.; 19 Division of Hospital Medicine, Mayo Clinic, Rochester, Minnesota, USA.; 20 Division of Infectious Diseases, The University of Texas Health Science Center at Houston, Houston, Texas, USA.; 21 Department of Internal Medicine, St. Joseph Mercy Hospital, Ann Arbor, Michigan, USA.; 22 Department of Medicine, Vanderbilt University Medical Center, Nashville, Tennessee, USA.; 23 Cook Children’s Health Care System, Fort Worth, Texas, USA.; 24 Department of Neurosurgery, Baylor College of Medicine, Houston, Texas, USA.; 25 Division of Infectious Diseases, Penn State Milton S. Hershey Medical Center, Hershey, Pennsylvania, USA.; 26 Division of Infectious Diseases, Mayo Clinic Hospital, Phoenix, Arizona, USA.; 27 NIAID, NIH, Bethesda, Maryland, USA.; 28 Department of Pathology, Thomas Jefferson University, Philadelphia, Pennsylvania, USA.; 29 Division of Infectious Diseases and Hospital Epidemiology, Cantonal Hospital St. Gallen, Switzerland.; 30 Department of Pathology, University of Texas Health San Antonio, San Antonio, Texas, USA.; 31 LCIM, NIAID, NIH, Bethesda, Maryland, USA.; 32 Wilmington Health, Wilmington, North Carolina, USA.; 33 Department of Internal Medicine, University of Texas Health Science Center, Houston, Texas, USA.; 34 Department of Pharmaceutical Sciences, School of Pharmacy, Texas Tech University Health Sciences Center, Amarillo, Texas, USA.; 35 Department of Pharmacy, University of Patras, Patras, Greece.; 36 Molecular Microbiology Section, LCIM and; 37 Metaorganism Immunity Section, Laboratory of Host Immunity and Microbiome, NIAID, NIH, Bethesda, Maryland, USA.; 38 Medical Research Council Centre for Medical Mycology, University of Exeter, Exeter, United Kingdom.; 39 Neutrophil Monitoring Laboratory, Applied/Developmental Research Directorate, Frederick National Laboratory for Cancer Research, Frederick, Maryland, USA.

**Keywords:** Immunology, Infectious disease, Fungal infections, Genetic variation, Innate immunity

## Abstract

Subcutaneous phaeohyphomycosis typically affects immunocompetent individuals following traumatic inoculation. Severe or disseminated infection can occur in CARD9 deficiency or after transplantation, but the mechanisms protecting against phaeohyphomycosis remain unclear. We evaluated a patient with progressive, refractory *Corynespora cassiicola* phaeohyphomycosis and found that he carried biallelic deleterious mutations in *CLEC7A* encoding the CARD9-coupled, β-glucan–binding receptor, Dectin-1. The patient’s PBMCs failed to produce TNF-α and IL-1β in response to β-glucan and/or *C. cassiicola*. To confirm the cellular and molecular requirements for immunity against *C. cassiicola*, we developed a mouse model of this infection. Mouse macrophages required Dectin-1 and CARD9 for IL-1β and TNF-α production, which enhanced fungal killing in an interdependent manner. Deficiency of either Dectin-1 or CARD9 was associated with more severe fungal disease, recapitulating the human observation. Because these data implicated impaired Dectin-1 responses in susceptibility to phaeohyphomycosis, we evaluated 17 additional unrelated patients with severe forms of the infection. We found that 12 out of 17 carried deleterious *CLEC7A* mutations associated with an altered Dectin-1 extracellular C-terminal domain and impaired Dectin-1–dependent cytokine production. Thus, we show that Dectin-1 and CARD9 promote protective TNF-α– and IL-1β–mediated macrophage defense against *C*. *cassiicola*. More broadly, we demonstrate that human Dectin-1 deficiency may contribute to susceptibility to severe phaeohyphomycosis by certain dematiaceous fungi.

## Graphical abstract

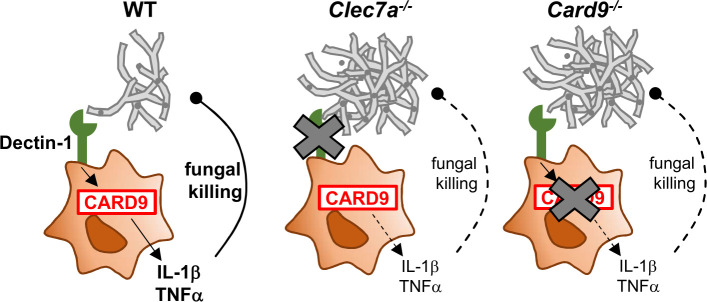

## Introduction

Phaeohyphomycosis is an invasive fungal infection caused by dematiaceous fungi, which are characterized by melanin production and filamentous growth. Phaeohyphomycosis typically affects the subcutaneous tissues following traumatic inoculation and is treatable with antifungal therapy and/or surgical resection. Phaeohyphomycosis may also manifest as cerebral or disseminated disease in immunosuppressed patients or, infrequently, in putatively immunocompetent individuals, and its prognosis is usually poor (1, 2). Better understanding of the pathogenesis of phaeohyphomycosis may improve the management and outcome of affected patients.

Phaeohyphomycosis has been reported in CARD9 deficiency (3–5), a primary immunodeficiency disorder characterized by severe fungal infections that involve predominantly the oral mucosa, subcutaneous tissues, and brain (5–13). CARD9 deficiency is caused by biallelic loss-of-function mutations in *CARD9*, which encodes an adaptor protein that relays fungus-sensing signals by multiple C-type lectin receptors (CLRs), such as Dectin-1 (6, 10, 14). Mechanistically, CARD9 mediates neutrophil effector function and recruitment to the fungus-infected brain, via IL-1β and CXCL1 production by brain-resident phagocytes (15–17). The CARD9-dependent mechanisms of antifungal protection within subcutaneous tissues remain poorly defined, with recent studies indicating that CARD9 may promote neutrophil recruitment to that tissue (3) and IL-17 production (18). CARD9-coupled CLRs, such as Dectin-1, have also been implicated in activating protective antifungal immune responses in humans. For example, the deleterious mutation p.Y238* in *CLEC7A,* which encodes Dectin-1, has been previously associated with familial vaginal yeast infections and onychomycosis, which was linked to impaired Dectin-1–dependent production of proinflammatory cytokines, including IL-17, in response to the Dectin-1 ligand, β-glucan, and *Candida* yeast cells (19). In addition, the presence of p.Y238* in either donors or recipients of allogeneic hematopoietic stem cell transplantation has been associated with the development of invasive pulmonary aspergillosis (20).

We describe an index patient with severe phaeohyphomycosis caused by *Corynespora cassiicola*, a dematiaceous fungus of plants known to cause life-threatening infections in CARD9 deficiency (4, 21). Our patient had biallelic deleterious mutations in *CLEC7A*, which encodes the CARD9-coupled receptor, Dectin-1. We show that Dectin-1 is critical for IL-1β and TNF-α production against this fungus by human immune cells. Both Dectin-1 and CARD9 deficiencies heightened susceptibility in a mouse model of subcutaneous *C*. *cassiicola* infection. Protection involved Dectin-1– and CARD9-dependent IL-1β and TNF-α production, which enhanced macrophage fungal killing. We also evaluated 17 additional unrelated patients with severe phaeohyphomycosis and found that 12 out of 17 had deleterious *CLEC7A* mutations, which were associated with an altered Dectin-1 extracellular, β-glucan–binding, C-terminal domain, and impaired Dectin-1–dependent cytokine production. Thus, human Dectin-1 deficiency may contribute to susceptibility to severe phaeohyphomycosis by certain dematiaceous fungi, likely following traumatic inoculation.

## Results

### Clinical presentation.

**Figure 1:**
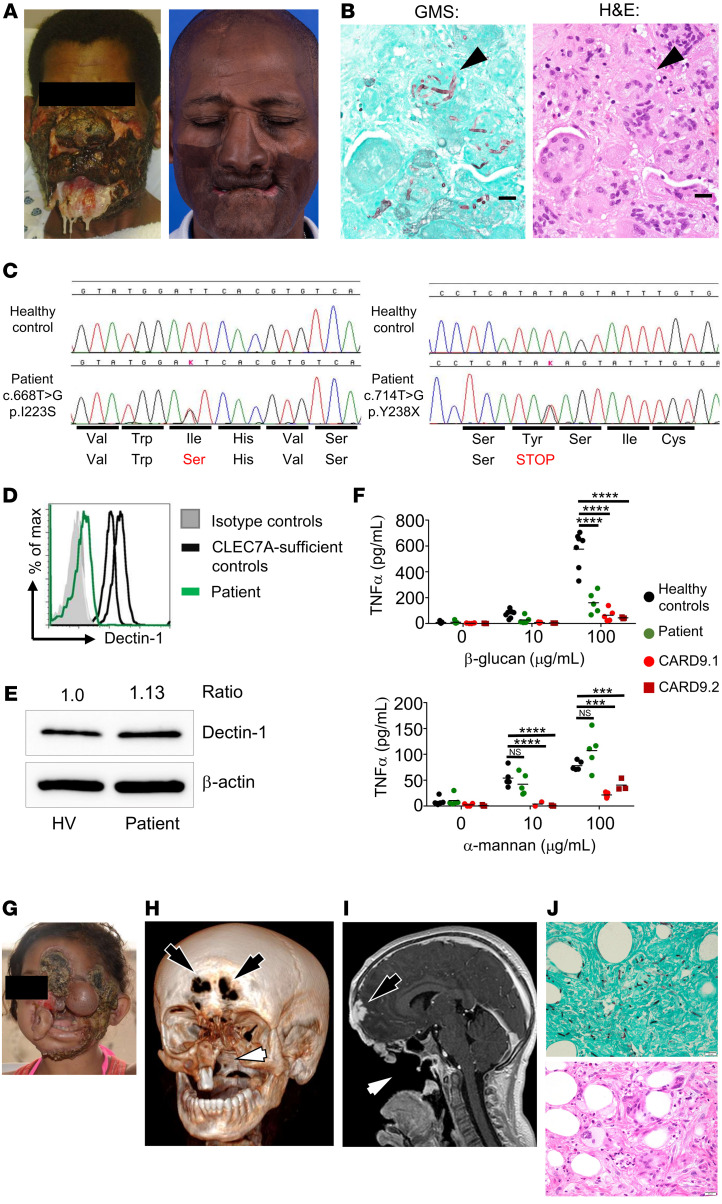
A Dectin-1–deficient patient with severe *Corynespora* *cassiicola* phaeohyphomycosis. (**A**) Photographs of the index patient at presentation at the NIH in 2004 (left) and following antifungal treatment and secondary prophylaxis in 2018 (right). (**B**) Grocott’s methenamine silver–stained (GMS-stained, left) and hematoxylin and eosin–stained (H&E-stained, right) section of soft tissue biopsy demonstrating *C*. *cassiicola* being engulfed but not destroyed in macrophages (black arrowheads) within granulomas (×100 magnification shown in Supplemental Figure 1). Scale bars: 20 μm. (**C**) Chromatograms from *CLEC7A* sequencing on healthy control and our patient, over the site of mutation in each allele. (**D**) Representative FACS histograms showing Dectin-1 surface expression in our patient and 2 healthy control patients. Histograms were gated on CD14^+^ monocytes isolated from peripheral blood. (**E**) Representative protein immunoblot images of Dectin-1 expression in PBMCs from our patient and a healthy volunteer (HV). β-Actin was used as loading control. (**F**) TNF-α production by PBMCs after 48 hours of stimulation with either purified particulate β-glucan or α-mannan. Each data point represents an individual well; at least 2 separate blood draws were analyzed in the Dectin-1–deficient patient (each tested in 2–3 technical replicates) and compared to 2 different healthy controls (each tested in 2–3 technical replicates). A single blood draw from each of the CARD9-deficient patients was analyzed in 3–5 technical replicates. Data in panel **F** were analyzed by 2-way ANOVA with Bonferroni’s correction. ***P < 0.005, ****P < 0.0001. NS, not significant. (**G**) Photograph of a previously reported CARD9-deficient patient (CARD9.02) (21) at presentation at the NIH (age 12). (**H**) Volume rendering of computed tomography data of patient CARD9.02 at age 12 emphasizing bone, which reveals erosions of the frontal bone (black arrows) and loss of maxillofacial structures (white arrow), including the hard palate, resulting in a common oronasal cavity. (**I**) Parasagittal T1-weighted magnetic resonance imaging of patient CARD9.02 at age 12 obtained following i.v. gadolinium-based contrast agent administration, which reveals epidural abscess with adjacent cerebritis (black arrow) and tissue loss (white arrow) resulting in a common cavity encompassing the nasopharynx, oropharynx, nasal cavity, oral cavity, and portions of the paranasal sinuses. (**J**) GMS- (upper) and H&E-stained (lower) section of soft tissue biopsy demonstrating granulomatous inflammation with *C*. *cassiicola* engulfed within macrophages (accompanying images from brain biopsy shown in Supplemental Figure 4). Both images are from consecutive cuts of the same biopsy sample. Scale bars: 20 μm.

A 64-year-old African American male living in Louisiana, United States, first presented to a local hospital in 1984 (at age 27) with a nonhealing growth on his nose following an injury he sustained while working in construction (22). Biopsy revealed mixed yeast and melanin-pigmented fungal filaments, leading to the diagnosis of phaeohyphomycosis, although identification at the fungal species level was not possible. The patient had a normal peripheral white blood cell count and differential and unremarkable chemistries. He was treated with oral ketoconazole (200 mg/day) for 9 months with some improvement, but the infection recurred within 6 weeks of stopping therapy. Three additional months of ketoconazole failed to improve the nasal lesion, prompting a dose increase to 400 mg for 4 more months, which led to marked improvement (22). However, the patient experienced several infection relapses over the subsequent 18 years. In 2004 (at age 47), he presented to an outside hospital with severe facial ulcerations. Despite escalation of antifungal therapy to itraconazole, amphotericin B, and caspofungin, and multiple debridements, the infection progressed, and the patient was transferred to the NIH Clinical Center. He had no other medical conditions and no prior history of infections, including mucocutaneous candidiasis, dermatophytosis, or onychomycosis. His family history was noncontributory. Physical examination revealed extensive ulcerative and necrotic facial lesions (Figure 1A). Laboratory studies revealed leukocytosis with a white blood cell count of 30,000 cells/μL, of which 89% were neutrophils, 2.8% monocytes, 7.7% lymphocytes, and 0.5% eosinophils. Serum immunoglobulin (including IgE) levels were within normal limits. Biopsy of the facial lesions demonstrated granulomatous inflammation with plasma cells, few neutrophils, and rare eosinophils, with fungal elements engulfed, but not destroyed, within macrophages (Figure 1B and Supplemental Figure 1; supplemental material available online with this article; https://doi.org/10.1172/JCI159348DS1). Fungi grew as sterile mycelia and were identified molecularly as *C*. *cassiicola* (Supplemental Figure 2). The minimal inhibitory concentrations for amphotericin B, itraconazole, posaconazole, caspofungin, and terbinafine were low at 0.125, 0.06, 0.06, 0.125, and 0.06 μg/mL, respectively. The facial lesions were extensively debrided. While receiving posaconazole and caspofungin, a cerebellar lesion was noted, and biopsy demonstrated neutrophilic microabscesses and granulomatous inflammation and fungal elements. 5-Flucytosine, terbinafine, and subcutaneous IFN-γ were added and the patient eventually improved. Multiple reconstructive surgeries and skin grafting procedures were performed over the subsequent year. The patient has not developed any recurrences over the past 16 years while receiving itraconazole secondary prophylaxis (Figure 1A).

### Identification of biallelic deleterious CLEC7A mutations.

A dihydrorhodamine test revealed normal phagocyte respiratory burst. We performed whole-exome sequencing to search for genes underlying human fungal susceptibility (23). We identified biallelic mutations in *CLEC7A* (Figure 1C and Table 1), which encodes Dectin-1. One allele contained the c.714T>G (p.Y238*) nonsense mutation (combined annotation-dependent depletion [CADD] score 36; https://cadd.gs.washington.edu/score Accessed January, 2021.) (19). When in homozygosity or heterozygosity, p.Y238* has been associated with impaired Dectin-1–dependent cellular responses (19, 20, 24, 25). The other allele had the missense mutation c.668T>G (p.I223S), which is predicted to be deleterious (PolyPhen-2 score 1.0 [http://genetics.bwh.harvard.edu/pph2/ Accessed January, 2021]; CADD score 23.8). The p.Y238* and p.I223S mutations have population allelic frequencies of 6.0% and 0.33%, respectively (gnomAD v2.1.1; https://gnomad.broadinstitute.org/ Accessed May, 2021).

We examined the consequences of these *CLEC7A* mutations for Dectin-1 expression. The patient’s monocytes had significantly reduced Dectin-1 surface expression by flow cytometry with an antibody that recognizes the extracellular, β-glucan–binding, C-terminal domain where the Dectin-1 mutations reside (Figure 1D and Supplemental Figure 3). By contrast, Dectin-1 expression in PBMCs was normal by immunoblot analysis with an antibody that recognizes the extracellular stalk region of Dectin-1, which is unaffected by the patient’s *CLEC7A* mutations (Figure 1E and Supplemental Figure 3; see complete unedited blots in the supplemental material). Normal Dectin-1 expression by immunoblot was verified with the antibody that recognizes the extracellular stalk region in HEK293 cells transfected with p.Y238* (Supplemental Figure 4; see complete unedited blots in the supplemental material). Therefore, p.Y238* does not decrease Dectin-1 protein production but it alters its extracellular β-glucan–binding C-terminal domain. To understand the functional consequences of these mutations, we stimulated PBMCs from the patient and healthy volunteers with purified β-glucan (a Dectin-1 ligand; ref. 26) or α-mannan (a Dectin-2 ligand; ref. 27) and measured TNF-α production, since phaeohyphomycosis is a reported complication of TNF-α–targeted biologics (28). The patient’s cells had blunted TNF-α production in response to β-glucan but not in response to α-mannan, which does not bind Dectin-1 (Figure 1F). As controls, PBMCs from 2 CARD9-deficient patients, one with invasive candidiasis (CARD9.1; ref. 16) and another with *C*. *cassiicola* phaeohyphomycosis (CARD9.2; ref. 21), did not respond to either β-glucan or α-mannan (Figure 1F).

Patient CARD9.2 initially developed subcutaneous *C*. *cassiicola* phaeohyphomycosis at age 4 in Colombia (21) and was admitted to the NIH Clinical Center at age 12. She had extensive, uncontrolled disease involving subcutaneous tissues of her face, adjacent bones, and brain (Figure 1, G–I). As with our index patient, biopsy of infected lesions demonstrated ineffective granulomatous inflammation, with fungal elements engulfed within macrophages (Figure 1J and Supplemental Figure 5). Over a 13-month hospital admission, the patient developed progressive fatal infection despite treatment with amphotericin B, posaconazole, micafungin, and terbinafine, repeated surgical debridements, and allogeneic hematopoietic stem cell transplantation. Thus, our index patient is compound heterozygous for *CLEC7A* mutations, which cause functional Dectin-1 deficiency associated with production of a protein with an altered extracellular C-terminal domain. Mechanistically, our Dectin-1–deficient patient is clinically and histologically similar and similarly defective in TNF-α production to a CARD9-deficient patient who developed fatal *C*. *cassiicola* phaeohyphomycosis.

### Dectin-1 binds and mediates proinflammatory cytokine responses to C. cassiicola.

**Figure 2:**
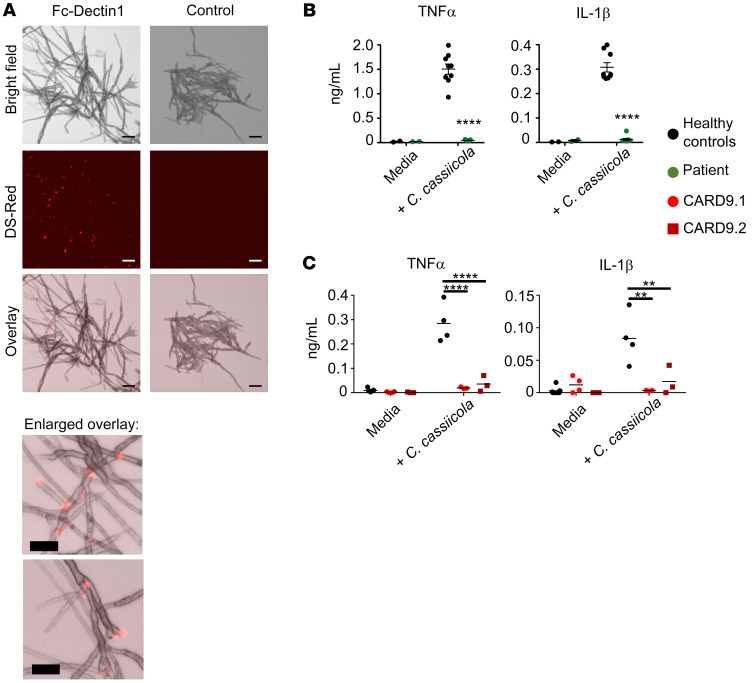
Dectin-1 binds to *Corynespora* *cassiicola* and contributes to proinflammatory cytokine production in response to the fungus. (**A**) Representative images of staining for Dectin-1–binding pathogen-associated molecular patterns using soluble Dectin-1 (Dectin-1 recognition domain fused with human Fc fragment) on *C*. *cassiicola*. Anti–human Fc–PE (visualized in DS-Red channel) was used as the secondary antibody or was used alone as negative control. Scale bars: 50 μm (upper panel) and 25 μm (enlarged images of *C*. *cassiicola* overlay in lower panel). (**B**) Cytokine production by PBMCs stimulated ex vivo with *C*. *cassiicola* in healthy controls (*n* = 2 donors, each tested in 3–4 technical replicates) and our patient (*n* = 2 different blood draws, tested in 3–4 technical replicates). (**C**) Shows similar experiments using PBMCs from 2 CARD9-deficient patients (1 blood draw per patient tested in 3–4 technical replicates), compared to 1 healthy donor tested in 4 technical replicates. Data in panels **B** and **C** were analyzed by 2-way ANOVA with Bonferroni’s correction. **P < 0.01, ****P < 0.0001.

We further investigated the immunological relevance of Dectin-1 deficiency in our index patient. We asked whether Dectin-1–sensing pathogen-associated molecular patterns were present on the *C*. *cassiicola* surface by staining with soluble Dectin-1 molecules (29). Indeed, *C*. *cassiicola* had Dectin-1 binding sites at filamentous branching points and leading ends, where conidia are released and bud scars form (Figure 2A).

We next examined the dependence on Dectin-1 for mounting proinflammatory cytokine responses to this fungus by stimulating PBMCs from the Dectin-1–deficient patient with *C*. *cassiicola*. PBMCs from CARD9-deficient patients and heathy volunteers served as controls. PBMCs from the Dectin-1–deficient patient produced significantly less TNF-α and IL-1β in response to *C*. *cassiicola* (Figure 2B), similar to CARD9-deficient PBMCs (Figure 2C). By contrast, responses to the Toll-like receptor (TLR) agonists LPS and Pam3CSK4 were normal, confirming selective impairment of antifungal responses (Supplemental Figure 6). Collectively, these data indicate that Dectin-1 binds and mediates proinflammatory cytokine responses to *C*. *cassiicola*.

### Dectin-1 and CARD9 deficiencies predispose to subcutaneous C. cassiicola phaeohyphomycosis in mice.

**Figure 3:**
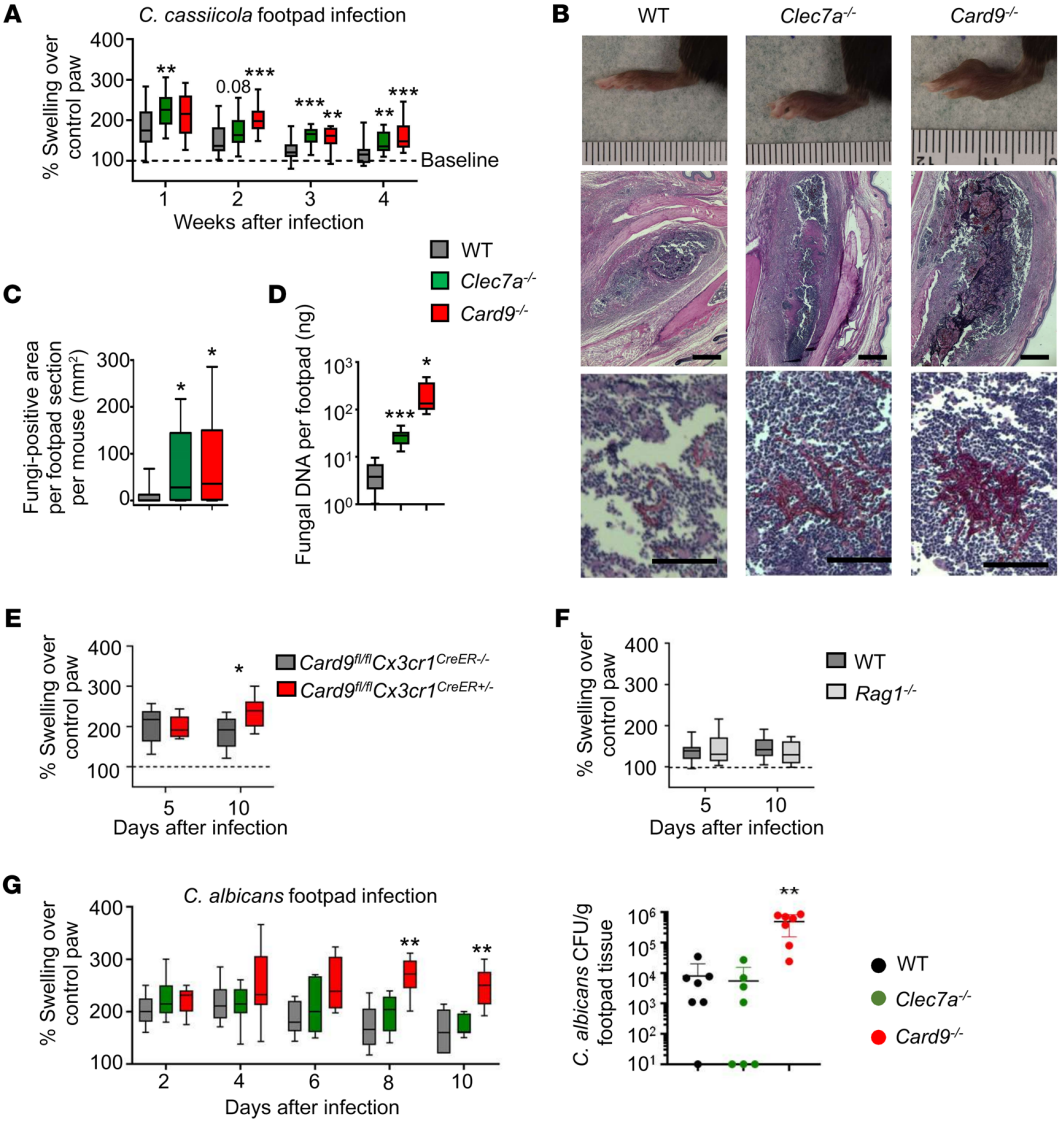
Dectin-1 and CARD9 deficiencies heighten infection susceptibility in a murine model of *Corynespora* *cassiicola* phaeohyphomycosis. (**A**) Footpad swelling after infection in WT (week 1 *n* = 27, week 2 *n* = 25, week 3 *n* = 20, week 4 *n* = 20), *Clec7a^–/–^* (week 1 *n* = 17, week 2 *n* = 18, week 3 *n* = 14, week 4 *n* = 14), and *Card9^–/–^* (week 1 *n* = 10, week 2 *n* = 10, week 3 *n* = 11, week 4 *n* = 10) mice. Data were pooled from 3 independent experiments and analyzed by 2-way ANOVA with Bonferroni’s correction. (**B**) Representative images of footpad swelling and histological analysis (on day 10 after infection) used to generate data shown in panel **C**, which is the area of footpad occupied by fungal cells on day 10 after infection (WT *n* = 10, *Clec7a^–/–^* *n* = 9, *Card9^–/–^* *n* = 7). Data in panel **C** were pooled from 3 independent experiments and analyzed using Mann-Whitney *U* test. Scale bars: 500 μm (**B**, second row) and 50 μm (**B**, third row). (**D**) *C*. *cassiicola* burdens in the footpad using qPCR-based quantification in WT (*n* = 6), *Clec7a^–/–^* (*n* = 8), and *Card9^–/–^* (*n* = 6) mice. Fungal DNA determined relative to standard curve of purified *C*. *cassiicola* genomic DNA (see Methods). Data were analyzed by 1-way ANOVA with Dunnett’s multiple comparison correction. (**E**) Footpad swelling in monocyte/macrophage-specific CARD9-deficient (*Card9^fl/fl^* *Cx3cr1^CreER+/–^*, *n* = 7) mice compared to their Cre-negative (CARD9-sufficient, *n* = 11) littermate controls. (**F**) Footpad swelling in *Rag1^–/–^* mice (*n* = 14) compared to their WT controls (*n* = 13). (**G**) Footpad swelling and fungal burdens in WT (*n* = 7), *Clec7a^–/–^* (*n* = 7), and *Card9^–/–^* (*n* = 7) mice infected in the hind footpad with 5 × 10^6^ CFU of *C*. *albicans* SC5314. Data in panels **E**–**G** were pooled from 2 independent experiments and analyzed by 2-way ANOVA with Bonferroni’s correction. Fungal burden data in panel **G** were analyzed by Mann-Whitney *U* test. *P < 0.05; **P < 0.01; ***P < 0.005.

Human CARD9 deficiency underlies progressive subcutaneous *C*. *cassiicola* phaeohyphomycosis (4, 21). We modeled subcutaneous *C*. *cassiicola* phaeohyphomycosis in mice to determine whether Dectin-1 is important for defense against this fungus in a controlled experimental setting. Following subcutaneous fungal inoculation, wild-type (WT) mice exhibited swollen footpads during the first week, which resolved over the next 2–3 weeks (Figure 3A). *Card9^–/–^* mice exhibited footpad swelling that was significantly greater than that of WT mice (Figure 3, A and B). We were unable to quantify live fungal organisms from the infected footpads by culture due to the mycelial growth of the fungus impeding CFU-based quantification. We therefore first used histological analysis, which revealed large granulomas with neutrophils surrounded by lymphocytes and histiocytes and significantly increased *C*. *cassiicola* invasion within granulomas of *Card9^–/–^* mice. The increased presence of fungal organisms was confirmed by fungal qPCR (Figure 3, B–D). Notably, *Clec7a^–/–^* mice also exhibited significantly greater footpad swelling, *C*. *cassiicola* invasion within granulomas, and footpad fungal burden by qPCR compared with WT mice, albeit to a lesser extent than *Card9^–/–^* mice (Figure 3, A–D). Therefore, Dectin-1 is necessary for defense against experimental *C*. *cassiicola* infection.

To define which immune cells mediate protective immunity during subcutaneous *C*. *cassiicola* phaeohyphomycosis, we infected monocyte/macrophage-specific CARD9-deficient *Card9^fl/fl^* *Cx3cr1^CreER^* mice (15) and found that CARD9 deficiency in monocytes/macrophages enhanced infection susceptibility relative to WT controls (Figure 3E). By contrast, *Rag1^–/–^* mice that lack lymphocytes were not susceptible (Figure 3F). Thus, monocyte/macrophage-dependent responses protect, while adaptive immunity is dispensable in this model.

Next, we examined the fungal specificity of the Dectin-1 and CARD9 dependence for defense during subcutaneous infection. Our patient did not manifest mucocutaneous candidiasis and *Clec7a^–/–^* mice control oropharyngeal candidiasis normally (30). During subcutaneous candidiasis, *Card9^–/–^* mice were susceptible, showing increased footpad swelling and fungal burden compared with WT mice. By contrast, *Clec7a^–/–^* animals controlled subcutaneous candidiasis similarly to WT (Figure 3G). Therefore, mouse Dectin-1 deficiency confers susceptibility to *C*. *cassiicola* but not *Candida* *albicans* subcutaneous infection, reflecting the fungus-specific susceptibility of our Dectin-1–deficient patient to *C*. *cassiicola* infection.

### Dectin-1– and CARD9-dependent IL-1β and TNF-α contribute to protection during subcutaneous C. cassiicola phaeohyphomycosis.

**Figure 4:**
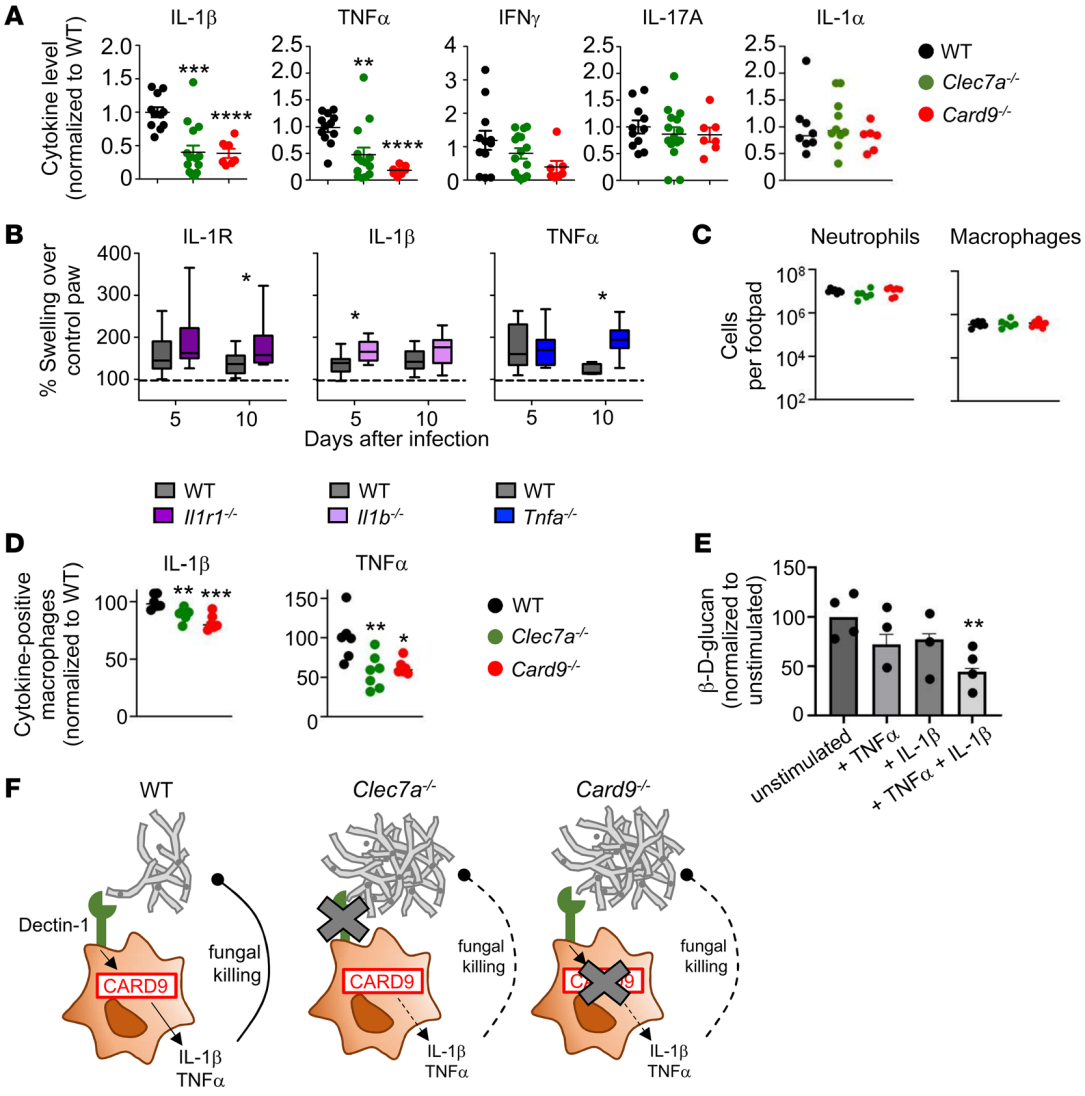
Dectin-1 and CARD9 promote TNF-α and IL-1β production during experimental *Corynespora* *cassiicola* phaeohyphomycosis that enhances macrophage *C*. *cassiicola* killing. (**A**) Cytokine analysis in the infected footpad homogenates (WT *n* = 12 mice, *Clec7a^–/–^* *n* = 12 mice, *Card9^–/–^* *n* = 7 mice) on day 3 after infection. Each data point represents an individual mouse; data were pooled from 2 independent experiments. (**B**) Footpad swelling in *Il1r^–/–^* (day 5: WT *n* = 24, KO *n* = 23, 3 pooled experiments; day 10: WT *n* = 16, KO *n* = 20, 4 pooled experiments), *Il1b^–/–^* (WT *n* = 7, KO *n* = 5, 1 experiment), and *Tnfa^–/–^* (day 5: WT *n* = 13, KO *n* = 15, 2 pooled experiments; day 10: WT *n* = 5, KO *n* = 15, 2 pooled experiments) mice, relative to WT controls. (**C**) Total numbers of neutrophils (live CD45^+^CD11b^+^Ly6G^+^) and macrophages (live CD45^+^CD11b^+^MHCII^hi^F4/80^+^) in the infected footpad on day 3 after infection (*n* = 6 mice per group), measured using flow cytometry. (**D**) IL-1β and TNF-α production within footpad macrophages on day 3 after infection in *Clec7a^–/–^* (*n* = 7) and *Card9^–/–^* mice (*n* = 6), normalized to the WT controls (*n* = 6). Each data point represents an individual mouse; data were pooled from 2 independent experiments and analyzed by 1-way ANOVA with Dunnett’s correction. (**E**) Results of an in vitro *C*. *cassiicola* killing assay with bone marrow–derived macrophages, prestimulated for 24 hours with TNF-α, IL-1β, or both. Killing was determined by measuring β-D-glucan levels in the culture supernatant. Bar graph shows the mean ± SEM for 2 independent experiments; overlaid dot plot shows technical replicates from one of these experiments. (**F**) Schematic representation of the proposed model of anti–*C*. *cassiicola* immunity in the footpad of WT, Dectin-1–deficient, and CARD9-deficient mice. Data in panels **A** and **B** were analyzed by 2-way ANOVA with Bonferroni’s correction. Data in panel **E** were analyzed by unpaired, 2-tailed *t* test. *P < 0.05; **P < 0.01; ***P < 0.005; ****P < 0.0001.

We next asked which cytokines protect during *C*. *cassiicola* phaeohyphomycosis and their dependence on Dectin-1/CARD9 signaling for production in vivo. We focused on IL-1α, IL-1β, TNF-α, and IFN-γ, whose production was abrogated in our index patient’s PBMCs upon *C*. *cassiicola* stimulation, and on IL-17A, which was produced normally by the patient’s PBMCs but promotes mucocutaneous antifungal immunity in other settings (Figure 2B and Supplemental Figure 7) (31, 32). The levels of IL-1α, IFN-γ, and IL-17A were not decreased within infected footpad homogenates of *Clec7a^–/–^* and *Card9^–/–^* mice relative to WT mice (Figure 4A). Although IFN-γ levels were similar, we examined its role further since recombinant IFN-γ had been administered as part of our index patient’s successful treatment. We found normal IFN-γ production by CD4^+^ T cells and intact expression of IFN-γ receptor 1 (IFN-γR1) by myeloid cells in the footpads of *Clec7a^–/–^* and *Card9^–/–^* mice relative to WT mice (Supplemental Figure 8, A and B). Moreover, we found similar fungal burdens in WT and *Ifng^–/–^* mouse footpads (Supplemental Figure 8C), collectively indicating that IFN-γ is dispensable for control of *C*. *cassiicola* infection in this model. These data are consistent with the lack of reported phaeohyphomycosis in patients with inherited IFN-γR deficiencies, neutralizing autoantibodies against IFN-γ, or receiving the IFN-γ–targeting monoclonal antibody emapalumab (23, 33, 34).

By contrast, the levels of IL-1β and TNF-α were significantly decreased within infected footpad homogenates of *Clec7a^–/–^* and *Card9^–/–^* mice compared with WT mice (Figure 4A). To determine whether decreased IL-1β and TNF-α production during infection may contribute to the impaired protection seen in *Clec7a^–/–^* and *Card9^–/–^* mice, we infected IL-1 receptor type 1– (IL-1R1–), IL-1β–, and TNF-α–deficient mice and found them to exhibit increased footpad swelling after *C*. *cassiicola* infection (Figure 4B). Collectively, these data indicate that IL-1β and TNF-α contribute to protection during experimental *C*. *cassiicola* phaeohyphomycosis, and their production depends on Dectin-1/CARD9 signaling in mice and humans.

### IL-1β and TNF-α enhance macrophage killing of C. cassiicola.

Since macrophages, IL-1β, and TNF-α contribute to anti–*C*. *cassiicola* defense, we examined how these cytokines may promote protective macrophage-dependent immunity. Macrophage accumulation did not differ in *C*. *cassiicola*–infected subcutaneous tissue between WT, *Clec7a^–/–^*, and *Card9^–/–^* mice (Figure 4C). Similarly, Dectin-1 and CARD9 were dispensable for neutrophil accumulation in *C*. *cassiicola*–infected subcutaneous tissues (Figure 4C). Thus, CARD9 promotes neutrophil recruitment in a fungus- and tissue-specific manner, as it mediates neutrophil recruitment in *C*. *albicans*–infected brain and *Phialophora*-infected subcutaneous tissues (15, 18). Notably, although macrophage accumulation was unaffected, macrophage production of TNF-α and IL-1β was significantly decreased in the absence of Dectin-1 or CARD9 in *C*. *cassiicola*–infected subcutaneous tissue (Figure 4D). We hypothesized that TNF-α and IL-1β directly enhance the fungal killing capacity of macrophages, since these cytokines are known to enhance macrophage antimicrobial killing pathways (35), and we had observed poor containment of fungal growth within macrophages in the soft tissue biopsy of our index patient (Figure 1B and Supplemental Figure 1). Indeed, stimulation of macrophages with both IL-1β and TNF-α, but not either cytokine alone, significantly increased *C*. *cassiicola* killing compared with unstimulated cells (Figure 4E). Of note, macrophage killing of *C*. *cassiicola* did not depend on ROS, since blocking the production of ROS did not impair macrophage killing of the fungus in vitro (Supplemental Figure 9). Together, our data support a model in which Dectin-1–mediated *C*. *cassiicola* recognition by CARD9-expressing macrophages promotes TNF-α and IL-1β production, which enhances macrophage nonoxidative fungal killing during experimental *C*. *cassiicola* phaeohyphomycosis (Figure 4F).

### Deleterious CLEC7A mutations associated with impaired Dectin-1 responses are frequent in patients with severe phaeohyphomycosis.

**Figure 5:**
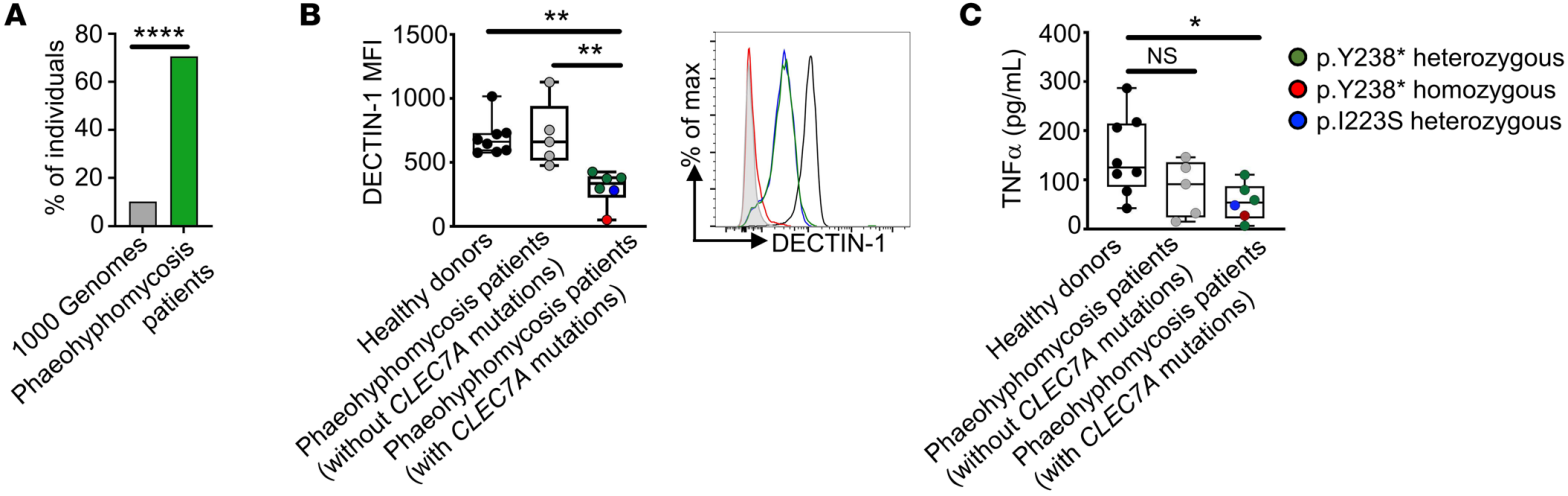
Deleterious *CLEC7A* mutations associated with impaired Dectin-1 responses are frequent among patients with severe phaeohyphomycosis. (**A**) Relative frequency of deleterious *CLEC7A* mutations with high CADD scores (>20) at the population level (calculated using data from 1000 Genomes) and in the index patient and 17 additional unrelated patients with severe forms of phaeohyphomycosis who were enrolled consecutively over an 8-year period at the NIH (see Table 1 for details). Data were analyzed by Fisher’s exact test. ****P < 0.0001. (**B**) Mean fluorescence intensity (MFI) values for Dectin-1 surface expression in CD14^+^ monocytes isolated from 8 healthy donors (black dots, see Supplemental Table 1 for details), 5 phaeohyphomycosis patients without *CLEC7A* mutations (gray dots), and 6 phaeohyphomycosis patients carrying *CLEC7A* mutations (green, red, and blue dots as indicated). FACS staining was performed using an antibody that targets the C-terminus where the *CLEC7A* mutations reside. No PBMCs were available for testing in the other patients with severe phaeohyphomycosis, including the one carrying the c.547C>T (p.Leu183Phe) variant (Table 1). FACS histograms show representative Dectin-1 staining for healthy control (black line), a patient homozygous for p.Y238* (red line), and patients heterozygous for p.Y238* (green line) or p.I223S (blue line) relative to isotype staining control (filled gray histogram). (**C**) TNF-α production by PBMCs stimulated with particulate β-glucan for 48 hours, from the same patients as shown in panel **B**. One blood draw was tested per healthy donor or patient in 3–12 technical replicates and the mean value per individual is depicted. Data were analyzed by 1-way ANOVA with Dunnett’s correction. *P < 0.05, **P < 0.01. NS, not significant.

To examine the potential broader implications of impaired Dectin-1 responses in human phaeohyphomycosis, we evaluated 17 other unrelated, putatively immunocompetent individuals with severe forms of phaeohyphomycosis who were enrolled consecutively over an 8-year period at the NIH Clinical Center. We found *CLEC7A* mutations with high CADD scores (>20) in 12 of them (70.9%) (Table 1). Combined with our index patient with *C*. *cassiicola* infection, 13 out of 18 (72.2%) patients with severe phaeohyphomycosis carried deleterious *CLEC7A* mutations: 5 with biallelic and 8 with heterozygous *CLEC7A* mutations. This represents a significantly greater frequency than that seen at the population level, evidenced by comparing to healthy individuals recorded in 1000 Genomes (https://www.internationalgenome.org/ Accessed January, 2021) (263 out of 2,504, 10.5%; *P* < 0.0001, Fisher’s exact test) (Figure 5A). Moreover, we found significantly reduced binding of the C-terminus–targeted anti–Dectin-1 antibody on the surface of monocytes and decreased TNF-α production upon β-glucan stimulation by PBMCs of patients with phaeohyphomycosis carrying *CLEC7A* mutations, consistent with impaired Dectin-1–dependent responses in these patients (Figure 5, B and C). Impaired TNF-α production was observed across Dectin-1 p.Y238* homozygous (red symbols) and heterozygous (green symbols) and Dectin-1 p.I223S heterozygous (blue symbols) patient PBMCs (Figure 5C), similar to the previously reported decreased proinflammatory cytokine production in heterozygous and homozygous Dectin-1 p.Y238* PBMCs (19, 20, 36–38), suggesting that p.Y238* and p.I223S may act in a dominant-negative manner. Collectively, these data indicate that impaired Dectin-1 responses may have contributed to severe phaeohyphomycosis in more than 50% of patients in our cohort with this infection.

## Discussion

We report a patient with Dectin-1 deficiency with severe *C*. *cassiicola* phaeohyphomycosis, an infection previously reported in inherited CARD9 deficiency. Therefore, Dectin-1 deficiency may phenocopy CARD9 deficiency with respect to susceptibility to this dematiaceous fungus in the setting of traumatic fungal inoculation. We show that Dectin-1 binds to *C*. *cassiicola* and promotes proinflammatory cytokine production by human PBMCs. In *C*. *cassiicola*–infected mice, Dectin-1 and CARD9 deficiencies impaired fungal control and decreased production of TNF-α and IL-1β, which contribute to protective immunity via macrophage fungal killing. Moreover, in an additional 12 out of 17 unrelated patients with severe forms of phaeohyphomycosis, we identified heterozygous or biallelic deleterious *CLEC7A* mutations associated with an altered Dectin-1 extracellular β-glucan–binding domain, impairing TNF-α production in response to fungal stimulation. Collectively, these data underscore the importance of Dectin-1 in antifungal immunity to phaeohyphomycetes.

*C. cassiicola* causes multiple diseases in plants (39). It also rarely causes human phaeohyphomycosis, affecting diabetic (40), CARD9-deficient (4, 21, 41), and apparently immunocompetent patients (42–44). Human Dectin-1 deficiency caused by p.Y238* homozygosity was previously associated with vulvovaginal candidiasis and onychomycosis (19). This susceptibility was linked to impaired Dectin-1–mediated recognition of *Candida* and decreased production of IL-17A and TNF-α (19). The p.Y238* mutation has also been associated with increased risk of mucosal *Candida* colonization and invasive aspergillosis in the setting of allogeneic hematopoietic stem cell transplantation, linked to impaired production of proinflammatory cytokines by PBMCs of heterozygous and homozygous patients (20, 38). We show here that whereas p.Y238* Dectin-1 protein is produced at adequate levels, it has an altered extracellular, β-glucan–binding, C-terminal domain, which could affect the innate recognition of *C*. *cassiicola*. We found that the C-terminal domain of Dectin-1 bound *C*. *cassiicola* at branching buds and hyphal tips, representing one of the first insights to our knowledge into innate recognition of this specific fungus. Whether other CARD9-coupled CLRs, TLRs, or other pattern recognition receptors can engage ligands within the *C*. *cassiicola* cell wall and initiate protective immunity, either alone or collaboratively, remains to be determined. Based on our data, we propose that human Dectin-1 deficiency contributes to severe *C*. *cassiicola* phaeohyphomycosis, and that deleterious *CLEC7A* (and *CARD9*) mutations should be sought in affected patients.

The rarity of *C*. *cassiicola* phaeohyphomycosis is notable relative to the frequency of human Dectin-1 deficiency, as approximately 5 and approximately 0.1 out of 1,000 individuals are homozygous for p.Y238* and p.I223S, respectively. Therefore, these deleterious *CLEC7A* variants in themselves do not represent a primary immunodeficiency per se. By contrast, these population *CLEC7A* variants are relatively benign unless the carrier is traumatically exposed to *C*. *cassiicola* or certain other phaeohyphomycetes to instigate disease, after which impaired Dectin-1–dependent immune responses appear to contribute to suboptimal infection control. The contribution of common population variants to infection susceptibility has been previously shown. For example, common heterozygous *CFTR* mutations are associated with increased risk of respiratory infections (45, 46). Furthermore, the common homozygous CCR5Δ32 mutation (~1% of Whites) increases the risk of severe West Nile virus infection upon exposure to the virus by mosquitos (47–49). Moreover, the common homozygous TYK2 P1104A mutation (~1 out of 600 of Europeans) increases the risk of tuberculosis upon exposure to *Mycobacterium* *tuberculosis* (50). Indeed, most cases of human *C*. *cassiicola* infections are reported to develop in the setting of physical injuries, as with our index patient (42). In addition, the low virulence of *C*. *cassiicola* associated with its marked growth restriction at temperatures greater than 30°C (39) and its preferential habitat on crop plants and debris (51) further limits the available opportunities for this fungus to cause human infection, and thus may additionally explain the rarity of this infection despite the relatively high frequency of deleterious *CLEC7A* variants in the general population. Of note, these deleterious *CLEC7A* mutations are enriched in the San population of South Africa, a region where subcutaneous mycoses are common (19, 52). Whether genetic variation in the CLR/CARD9 pathway may explain, at least partly, the increased prevalence of subcutaneous mycoses in certain subtropical regions warrants investigation. Our findings, together with the accompanying report by Hsu and colleagues (53), imply that Dectin-1 may be critical to the optimal development of antifungal defense following traumatic inoculation by low-virulence, dematiaceous fungi (*Corynespora*), or inhalational exposure to highly virulent fungi in certain geographic areas (*Coccidioides*).

To study the immunopathogenesis of *C*. *cassiicola* phaeohyphomycosis, we modeled it in mice. We used footpad swelling as a surrogate measure of infection control, which we found correlated to tissue fungal burden, although differential swelling in different gene-deficient mice may also be due to inappropriate inflammation and inability to form or resolve granulomas. Using this model, we found that Dectin-1 and CARD9 were each required for anti–*C*. *cassiicola* defense, which depended on macrophages, and TNF-α and IL-1β were critical for protection and macrophage fungal killing. The mechanism by which macrophages mediate *C*. *cassiicola* killing remains to be determined, although our data indicated that ROS was either not involved or could be compensated by additional nonoxidative mechanisms. These data are consistent with the normal oxidative burst of our index patient and the lack of reported phaeohyphomycosis in patients with chronic granulomatous disease who lack phagocyte oxidative burst (34, 54). Moreover, future studies will be required to determine whether human monocyte-derived macrophages also rely on TNF-α and IL-1β in an interdependent manner for anti–*C*. *cassiicola* defense. By contrast, neutrophil accumulation was unaffected by Dectin-1 and CARD9 deficiency in this model, although we cannot rule out functional deficits in these cells or that reduced IL-1β and TNF-α may have had an effect on fungal uptake and killing by neutrophils; these questions will require further study using neutrophil-specific Dectin-1– and CARD9-deficient mice. However, we found that monocyte/macrophage-specific CARD9-deficient mice had enhanced susceptibility to *C*. *cassiicola* infection, indicating that macrophage defects are the primary driver of the disease in this model. In addition to intact neutrophil recruitment, we also found that IL-17A production within *C*. *cassiicola*–infected footpads were Dectin-1 and CARD9 independent. This contrasts with *Phialophora* and *Exophiala* phaeohyphomycoses, where neutrophil recruitment and IL-17A production were shown to be CARD9 dependent (3, 18). Thus, CLR/CARD9-dependent protection against phaeohyphomycosis may utilize differential fungal species–specific mechanisms, which merit further investigation. Future studies should decipher potential additional Dectin-1– and CARD9-dependent anti–*C*. *cassiicola* protective cellular and molecular mechanisms and define which Dectin-1–independent CARD9-dependent CLRs may also participate in defense against *C*. *cassiicola* and other dematiaceous fungi in mice and humans. Of note, a recent report indicated that impaired Dectin-2–dependent immune responses may have contributed to the development of fatal invasive aspergillosis in an immunosuppressed patient (55). Additional studies will be required to examine the mechanisms by which p.Y238* and p.I223S may act in a dominant or co-dominant manner and to model and study phaeohyphomycosis caused by *Cladophialophora*, *Bipolaris*, *Curvularia*, and other melanin-bearing agents of phaeohyphomycosis in WT, Dectin-1–deficient, CARD9-deficient, and heterozygous and homozygous p.Y238* and p.I223S mice. Moreover, the identification of deleterious *CLEC7A* mutations in approximately 70% of patients with severe phaeohyphomycosis in our cohort requires validation in future studies with more patients manifesting various fungus- and tissue-specific forms of severe phaeohyphomycosis. Furthermore, the role of prolonged antifungal therapy and/or long-term secondary antifungal prophylaxis in patients with severe phaeohyphomycosis who carry deleterious *CLEC7A* mutations requires further study.

In summary, we show that Dectin-1 deficiency may contribute to susceptibility to severe phaeohyphomycosis by certain dematiaceous fungi upon traumatic inoculation. Our findings provide mechanistic insights into the pathogenesis of phaeohyphomycosis and may help improve the diagnosis and management of immunocompetent patients who develop this infection.

## Methods

### Whole-exome sequencing.

Sequencing libraries were generated using the TruSeq DNA Sample Prep Kit (Illumina), following the NimbleGen SeqCap EZ Library SR User’s Guide, v4.0 (Roche Nimblegen, Inc.). Briefly, 1 μg of each gDNA was sheared using the Covaris instrument and Covaris microTUBEs (Covaris, Inc.). The following shearing conditions were used: duty cycle, 10%; intensity, 5.0; bursts per second, 200; duration, 120 seconds; mode, frequency sweeping; temperature, 5.5°C to 6.0°C. Each sample was prepared with a specific indexing adapter to facilitate multiplex pooling in the exon enrichment procedure. Exome enrichment was performed using the SeqCap EZ Human v3.0 Exome Enrichment kit on 200 ng of each TruSeq library. Libraries were combined to create a 6-plex reaction for the enrichment, yielding a total DNA library mass of 1,200 ng. One microgram of the 6-plex pooled libraries was hybridized to capture target oligonucleotides for 48 hours, as specified in the manufacturer’s protocol.

Exome-enriched libraries were quantified using the KAPA Library Quantification Kit (KAPA Biosystems), clustered on the cBot Cluster Station, and sequenced as 2 × 100 bp reads on the HiSeq 2500 instrument, according to the manufacturer’s protocol (Illumina).

High-quality, trimmed paired-end sequence reads were mapped to the human genome reference consortium GRGh37 (hg19) using Bowtie 2 and the default parameters with mixed mode disabled (57). Multiply mapped reads and PCR duplicates were removed using SAMtools (sequence alignment/map) (57). Additional filtering of the prealigned BAM files to remove reads with low-quality base, mapping, and alignment scores was performed prior to variant detection using Strand NGS 2.1 software (Strand Genomics, Inc.) and the following parameters: mapping quality threshold 20 or greater, base quality 17 or greater, and alignment score 85 or greater. Aligned reads were also base quality score recalibrated and locally realigned around indels. Reads were filtered against the SeqCap EZ Exome v3.0 target region (64 Mb exonic sequences) and reads greater than 100 bp outside of the targeted region were excluded from further analyses. The average coverage depth was 75× from approximately 1.9 Gb. Identification of SNPs and indels was performed using Strand NGS software. Strand NGS utilizes a modified Bayesian variant calling method adapted from the MAQ SNP calling algorithm which compares the nucleotides present on aligned reads against the reference at each position in the genome. The dbGaP study accession numbers are phs001899.v2.p1 and phs001561.

### CLEC7A sequencing.

Genomic DNA was isolated from peripheral blood leukocytes using a Gentra Puregene DNA isolation kit (Qiagen) and amplified using Platinum Taq PCR SuperMix High Fidelity (Thermo Fisher Scientific) following the manufacturers’ protocols with primers and cycling conditions listed in Supplemental Table 2. PCR products were purified with Exo-SAP IT (Thermo Fisher Scientific) and sequenced using BigDye Terminators v3.1 (Applied Biosystems) per the manufacturers’ instructions using primers listed in Supplemental Table 3. Reactions were purified over Performa DTR plates (Edge Biosystems) and resulting products run on an ABI 3730XL capillary sequencer. Chromatograms were analyzed using Sequencher (GeneCodes) and compared to the NCBI reference sequence, NM_197947.

### C. cassiicola culture.

The *C*. *cassiicola* strain used in all experiments was isolated from our Dectin-1–deficient patient. *C*. *cassiicola* was stored in 50% glycerol at –80°C, and routinely grown on potato dextrose agar (PDA; Sigma-Aldrich) plates at 20°C for 7 days, harvested, and replated onto fresh PDA plates and grown for an additional 7 days before use. To harvest *C*. *cassiicola*, 10 mL sterile tissue-culture grade water (Gibco) was added to each plate, and a soft paint brush (sterilized in 70% ethanol and rinsed in sterile water) used to brush the colonies and lift fungal cells. The fungi and water were pipetted off the plate, and a further wash/brush repeated with 5 mL of water. The resulting suspension was centrifuged (1,800*g* for 4 minutes) to pellet *C*. *cassiicola*, which was resuspended in sterile PBS. Each suspension was adjusted to an OD_600_ of 2.0 for use in experiments. Microscopy analysis and analysis of Dectin-1 ligand exposure was performed as outlined below.

### Dectin-1 ligand staining.

*C*. *cassiicola* was prepared as above and 50 μL used for staining. Pelleted fungal cells were resuspended in 1 mL 2% paraformaldehyde and incubated at room temperature for 10 minutes. Fungi were centrifuged again and supernatants discarded, before washing in 1 mL PBS. Cell pellets were resuspended in 400 μL 10% mouse serum/PBS and incubated for 15 minutes at room temperature to block, and then washed in 1 mL PBS. Cells were resuspended in 100 μL PBS and 1 μL Fc-Dectin-1 (29) added to the cells, incubating at 4°C for 1 hour with continual slow rotation. Fungi were washed twice in PBS, and then stained with 2 μg/mL anti–human IgG–PE secondary in 100 μL PBS for 30 minutes at 4°C. Cells were washed twice in PBS, resuspended in 20 μL PBS, added to microscope slides, and analyzed with a Leica fluorescence microscope equipped with ZEN software. *C*. *albicans* germ tubes (induced in 20% FBS/water for 1 hour at 37°C) were used as a positive control, and secondary-only–stained samples used as a negative control.

### Preparation of PBMCs.

PBMCs from the Dectin-1–deficient patients, CARD9-deficient patients, or healthy donors, were harvested from whole blood by gradient centrifugation using Lymphocyte Separation Media (Lonza), according to the manufacturer’s instructions. PBMCs were washed in PBS and resuspended in 10% DMSO/FBS, and slowly frozen before storing in liquid nitrogen. Frozen PBMCs were defrosted in a 37°C water bath and washed in RPMI 1640 supplemented with 10% FBS, 100 U/mL penicillin, and 100 μg/mL streptomycin (hereafter referred to as “complete RPMI”) prior to counting with trypan blue exclusion. Washed PBMCs were used for stimulation assays or flow cytometry.

### PBMC stimulation assay.

PBMCs (5 × 10^5^) were incubated in a round-bottom 96-well plate (Corning) at 37°C in a 5% CO_2_ incubator in complete RPMI, supplemented with either 200 ng/mL LPS (Sigma-Aldrich), 1 μg/mL Pam3CSK4 (Sigma-Aldrich), α-mannan (see below), β-glucan (see below), or heat-killed (incubation at 70°C for 1 hour) *C*. *cassiicola*, which was added at a 1:12 dilution to the cells. After 48 hours, PBMCs were pelleted and the supernatant was collected and stored at –80°C until analysis. Cytokine levels were analyzed via conventional ELISA (Figure 5B; TNF-α DuoSet, R&D Systems, DY210) or Luminex (Figure 1F). Luminex analysis was done via a multiplex bead array assay with antibodies and cytokine standards (R&D Systems, Peprotech). Individual Luminex bead sets (Luminex) were coupled to cytokine-specific capture antibodies according to the manufacturer’s protocols and biotinylated polyclonal antibodies were used at twice the recommended concentrations for a classical ELISA according to the manufacturer’s instructions. The assay was run with 1,200 beads per set of cytokines in a volume of 50 μL. The plates were read on a Luminex MAGPIX platform where more than 50 beads were collected per bead set. The median fluorescence intensity of the beads was then measured for each individual bead, which was analyzed with the Millipex software using a 5P regression algorithm.

### β-Glucan and α-mannan preparations.

Carbohydrates were purified as described previously (see main text for references), and then prepared for PBMC stimulation. In brief, 5 mg of particulate β-glucan was added to 1 mL water, sonicated for 30 seconds, pelleted by centrifugation at 10,000*g* for 10 minutes and resuspended in complete RPMI for use in stimulation experiments at the indicated concentrations. For α-mannan, 5 mg was dissolved in PBS, mixed at room temperature for 2 hours with shaking, centrifuged at 10,000*g* for 10 minutes, and resuspended in complete RPMI at indicated concentrations for stimulation experiments.

### Dectin-1 expression by flow cytometry.

PBMCs were isolated as above, resuspended in PBS, and Fc receptors blocked with anti-CD16/32 (BD Biosciences) on ice for 10 minutes prior to staining with anti–human CD14–FITC (Biolegend, clone HCD14) and anti–human Dectin-1–PE (Biolegend, clone 15E2), or the appropriate isotype control (Biolegend, IgG2a clone MOPC-173), on ice for 30 minutes. Stained samples were washed in PBS and acquired using a BD LSR, equipped with BD FACSDiva software. FlowJo (BD) was used for the final analysis.

### Dectin-1 immunoblot analysis.

Whole-cell lysates were suspended in 1× RIPA buffer containing protease and phosphatase inhibitors (Thermo Fisher Scientific). The lysates were centrifuged at approximately 14,000*g* for 15 minutes at 4°C. Supernatants were collected and protein concentrations were determined using the Bradford Protein Assay (Bio-Rad), according to the manufacturer’s protocol. The proteins (20 μg) were resolved in 12% SDS-PAGE and electrotransferred onto 0.2 μm PVDF. Membranes were blocked in 5% BSA and incubated with primary antibody against Dectin-1 (Invitrogen, PA5-34382) and β-actin (Cell Signaling Technology, clone D6A8), followed by secondary anti–rabbit IgG, HRP-linked antibodies (Cell Signaling Technology). Chemiluminescence detection was performed with Clarity Western ECL Blotting Substrate (Bio-Rad), using the ChemiDoc MP Imaging System (Bio-Rad). Quantification was obtained by densitometry image analysis using Image Lab 5.2 software (Bio-Rad).

### HEK293 transfection.

WT Dectin-1 expression plasmid (Origene, clone SC307610, NM_197947) was used for site-directed mutagenesis to generate the patient variant, c.714T>G; p.Y238*. HEK293 cells (ATCC) were plated at 5 × 10^5^ cells/well in a 6-well plate and allowed to rest overnight. The following day, cells were transfected using 2 μg WT or p.Y238* plasmid and Fugene HD reagent (Promega) at a 3:1 ratio. Twenty-four hours later, cells were harvested for immunoblot analysis.

### Mice.

Eight- to 12-week-old female mice were maintained in individually ventilated cages under specific pathogen–free conditions at the NIH. The following strains (and their respective WT controls/littermates) were obtained from the NIAID Taconic contract; *Rag1^–/–^*, *Il1r1^–/–^*, *Tnfa^–/–^*, and *Ifng^–/–^*. All other strains and their respective controls/littermates were bred in-house at the NIH (*Card9^–/–^*, *Clec7a^–/–^*, *Il1b^–/–^*, *Card9^fl/fl^* *Cx3cr1^Cre-ER+/–^*). In experiments using Cre-expressing mouse lines, mice were pretreated with 3 mg tamoxifen (dissolved in Miglyol 812, The Warner Graham Company) daily by intraperitoneal injection for 5 days prior to infection. Animals were infected and analyses performed as outlined below. Animals were euthanized by cervical dislocation following administration of ketamine/xylazine cocktail at defined time points after infection (see figure legends).

### Footpad infections.

*C*. *cassiicola* was prepared as above and 50 μL injected into the left hind footpad of each mouse using a 21-G needle. Footpad swelling was measured once per week using calipers, measuring the height of the foot just below the toes at the widest part of the foot. The right uninfected footpad was measured and swelling expressed as a percentage relative to the uninfected footpad for each individual mouse. For *C*. *albicans* footpad infections, *C*. *albicans* SC5314 was grown in YPD broth overnight at 30°C with shaking at 200 rpm, washed in PBS, and counted with a hemocytometer. Yeast cells (5 × 10^6^) were injected into the left hind footpad in a 50 μL volume and footpad swelling measured every 2 days as described above. *C*. *albicans* footpad burdens were measured by removing the infected footpad with a scalpel, homogenizing in 0.5 mL sterile PBS, and plating on YPD agar plates. Viable colonies were counted after incubation at 37°C for 24 hours and expressed as CFU per gram of footpad tissue.

### Footpad flow cytometry and intracellular cytokine staining.

Footpads were removed with a scalpel, finely minced, and digested for 30 minutes at 37°C in digestion medium (RPMI supplemented with 250 μg/mL Liberase TL and 50 μg/mL DNase [both from Roche]), which was supplemented with 10 μg/mL brefeldin A (Sigma-Aldrich) for intracellular cytokine staining experiments involving detection of IL-1β and TNF-α. The resulting cellular suspension was filtered and washed in PBS. Cells were stained with fluorophore-labeled antibodies against the following proteins: CD45 (Biolegend, clone 30-F11), Ly6G (Biolegend, clone 1A8), CD11b (Biolegend, clone M1/70), CD11c (eBioscience, clone N418), Ly6C (BD Biosciences, clone AL-21), MHCII (eBioscience, clone M5/114.15.2), F4/80 (Biolegend, clone BM8), CD3 (Biolegend, clone 145-2C11), TCRβ (Bioegend, clone H57-597), CD90.2 (eBioscience, clone 30-H12), CD4 (Biolegend, clone RM4-5), CD8 (Biolegend, clone 53-6.7), CD19 (Biolegend, clone 1D3), NKp46 (Biolegend, clone 29A1.4), and IFN-γR1 (eBioscience, clone 2E2), and data were acquired on a BD LSR Fortessa equipped with BD FACSDiva software. FlowJo (Tree Star) was used for the final analysis. For intracellular cytokine staining of IL-1β and TNF-α, mice were first injected with 250 μg brefeldin A 6 hours prior to sacrifice. To stain for IFN-γ^+^ cells, the cells were stimulated with PMA (50 ng/mL) and ionomycin (2.5 μg/mL) for 3.5 hours at 37°C and 5% CO_2_ in a humidified chamber, in the presence of 1 mg/mL brefeldin A. Cells were then fixed/permeabilized using the Foxp3 Staining Buffer Set (eBioscience) prior to labeling with anti–IL-1β (Invitrogen, clone NJTEN3), anti–TNF-α (Biolegend, clone MP6-XT22), or anti–IFN-γ (eBioscience, clone XMG1.2).

### C. cassiicola killing assay.

Bone marrow cells from the femurs/tibias of C57BL/6 females were flushed out with sterile 2 mM EDTA/PBS, washed, and resuspended in RPMI (with GlutaMAX; Invitrogen) with 20% FBS (Sigma-Aldrich), 1% penicillin/streptomycin (Invitrogen), and 40 ng/mL M-CSF (Biolegend). Bone marrow cells were incubated in 75 cm^2^ tissue culture flasks (Corning) for 5 days, replacing the media on day 3. On day 5, the media were replaced with ice-cold 2 mM EDTA/PBS and flasks incubated on ice for 10 minutes. Adherent cells (macrophages) were gently lifted using a cell scraper (Gibco) and counted using trypan blue exclusion. Macrophages were then seeded into flat-bottom 96-well plates (4 × 10^5^ per well) in RPMI supplemented with 10% FBS and 1% penicillin/streptomycin. Some wells were additionally supplemented with 10 ng/mL recombinant murine TNF-α (Biolegend) and/or 10 ng/mL recombinant murine IL-1β (Biolegend), or 5 mM *N*-acetyl cysteine (Sigma-Aldrich). Macrophages were incubated overnight at 37°C and 5% CO_2_. The following day, *C*. *cassiicola* (prepared as above to OD_600_ = 2.0) was added to the macrophages (50 μL fungal suspension added per well to a final volume of 200 μL) and the incubation at 37°C continued for a further 3 hours. Culture supernatants were then collected and frozen at –20°C. Concentrations of β-D-glucan in the supernatants were measured using the Glucatell (1→3)-β-D-glucan detection reagent kit (Cape Cod Associates), as per the manufacturer’s instructions.

### Footpad histology and cytokine determination.

The infected left hind foot of WT, *Card9^–/–^*, and *Clec7a^–/–^* mice was removed and fixed in 10% formalin for 24 hours before embedding in paraffin wax. Tissue sections were stained with periodic acid–Schiff (PAS). To estimate *C*. *cassiicola* burden in the footpad, pictures were taken of the entire footpad section (at least 2 per mouse) and the total area covered by fungal cells measured using ImageJ software (NIH). For cytokine analysis, infected footpads were removed with a scalpel and added to 0.5 mL PBS supplemented with 0.05% Tween 20 and a protease inhibitor cocktail (Roche). Footpads were homogenized and cell debris removed by centrifugation at 1,100*g* for 5 minutes at 4°C. The resulting supernatant was snap-frozen on dry ice and stored at –80°C until analysis. Samples were analyzed by Luminex array as described above, and cytokine concentrations were determined per milligram of footpad tissue.

### Determination of fungal burden in infected mouse footpads with qPCR.

On day 10 after infection, infected footpads of WT, *Card9^–/–^*, *Clec7a^–/–^*, and *Ifng^–/–^* mice were harvested and homogenized in 1 mL PBS using Omni tip homogenizers (Omni International). To isolate and quantify DNA from pure *C*. *cassiicola*, the fungus was grown as described above and the fungal suspension was adjusted to an OD_600_ of 2.0 in 1 mL PBS. The footpad homogenates and pure fungal suspensions were centrifuged at 5,000*g*, supernatants were discarded, and the pellets were utilized to isolate DNA as described previously (58). For qPCR, 20 ng of total DNA, PerfecTa Fast mix (Quanta Biosciences), fungus-specific Fungi-Quant probe/primers targeting the fungal *18s* rRNA gene, and specific cycling program were utilized as described previously (59). Fungal DNA amount per footpad was interpolated from a series of serially diluted DNA from pure *C*. *cassiicola*.

### Statistics.

Statistical analyses were performed using GraphPad Prism 9.0 software. Details of individual tests are included in the figure legends. In general, data were tested for normal distribution by the Kolmogorov-Smirnov normality test and analyzed accordingly by unpaired *t* tests or Mann-Whitney *U* test. In cases where multiple data sets were analyzed, either 2-way ANOVA was used with Bonferroni’s correction (animal experiments) or 1-way ANOVA with Dunnett’s multiple corrections (comparison of healthy controls and patients with/without Dectin-1 mutations for TNF-α production). See figure legends for details of tests used for each figure panel. To compare the frequency of deleterious *CLEC7A* mutations with high CADD scores (>20) in patients with severe phaeohyphomycosis relative to healthy individuals recorded in 1000 Genomes, Fisher’s exact was used. In all cases, *P* values of less than 0.05 were considered significant.

### Study approval.

Animal studies were performed in accordance with the recommendations in the NIH *Guide for the Care and Use of Laboratory Animals* (National Academies Press, 2011), under the auspices of protocol LCIM14E approved by the Animal Care and Use Committee of the NIAID. The patients and healthy donors were enrolled in protocols approved by the NIH Institutional Board Review and provided written informed consent for participation in the study. Patients or their parents provided written consent for their photographs to be published as part of this study and the record for the consent has been retained. This study was conducted in accordance with the Declaration of Helsinki. The Dectin-1–deficient index patient was enrolled in ClinicalTrials.gov study number NCT00001355. The CARD9-deficient patients and the additional putatively immunocompetent patients with severe forms of phaeohyphomycosis (Table 1) were enrolled in ClinicalTrials.gov study number NCT01386437. The 16 additional patients with severe forms of phaeohyphomycosis had not received iatrogenic immunosuppression (e.g., corticosteroids), were HIV negative, and had no prior history of fungal infections or other nonfungal infection susceptibility.

## Author contributions

RAD, JVD, APH, VO, JAA, SLA, MS, SV, DY, AK, NSPV, SP, AMZ, DBK, and MSL performed experiments and analyzed the data. RAD, JVD, APH, DCV, KDMB, AMZ, CMM, KJKC, JKL, DBK, SMH, and MSL designed experiments. MSA, MAW, DB, BD, KH, CSZ, EMNF, MMS, JAB, YRS, CAA, CA, MM, ZT, AGT, M Natarajan, M Nigo, VM, KCB, SGK, MSW, GR, CJW, NM, JGB, LCK, WRM, AG, AGJ, MS, AFF, JEB, SMH, KB, RH, and MSL provided clinical care and referred patients. AJO analyzed the genomic data. TD provided regulatory support to patient enrollment and consenting. KDMB, YI, KJKC, YB, and GDB provided key reagents and expertise. MSL conceived and supervised the project. RAD and MSL wrote the final manuscript.

## Supplementary Material

Supplemental data

## Figures and Tables

**Table 1:**
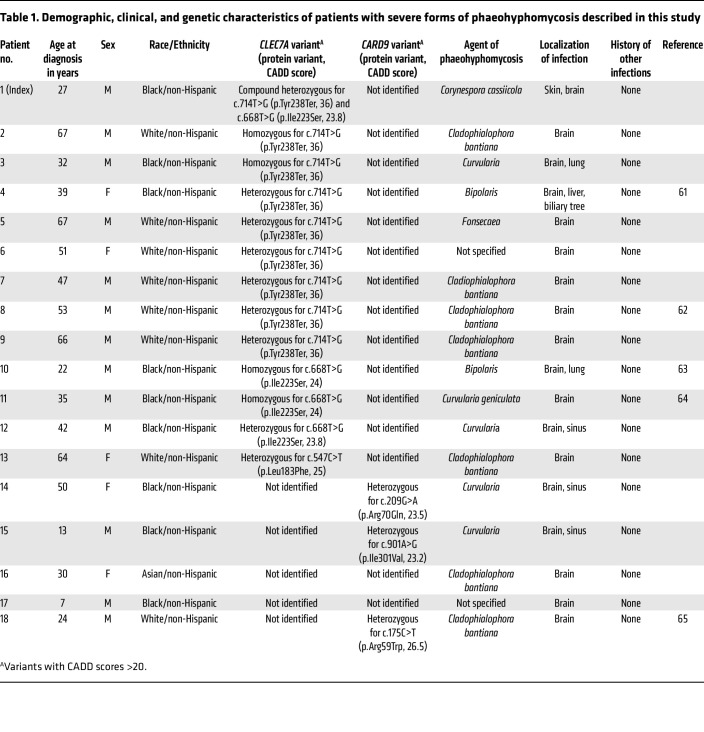
Demographic, clinical, and genetic characteristics of patients with severe forms of phaeohyphomycosis described in this study

## Version 1 11/15/2022

Electronic publication
