# Supplementary material for: Human Dectin-1 deficiency impairs macrophage-mediated defense against phaeohyphomycosis
Source: J Clin Invest. 2022 Nov 15;132(22):e159348. doi: 10.1172/JCI159348 (PMC9663159; doi:10.1172/JCI159348)

Supplemental Figures

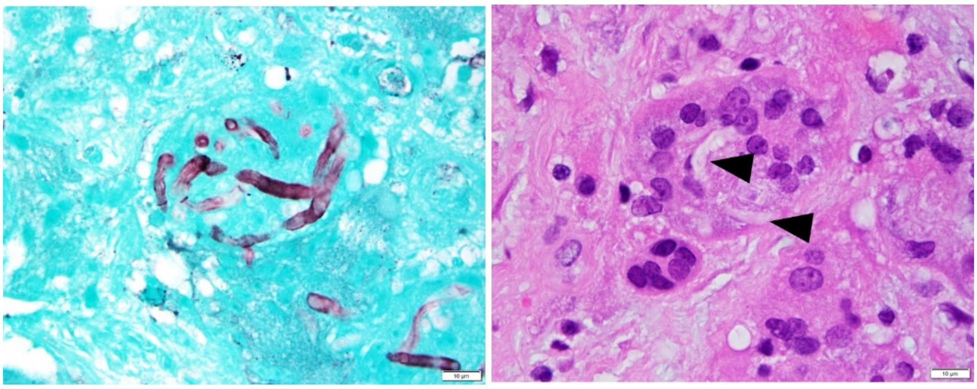

Figure S1: *C. cassiicola* hyphae are internalised but not destroyed within DECTIN-1-deficient macrophages. Histology of biopsy sample taken from our index patient (imaged at 100X magnification), stained with GMS (left) and H&E (right). Both images are from consecutive cuts of the same biopsy sample. Black arrows highlight fungal cells visible in the H&E image. The GMS-stained image (left) is a crop of the GMS-stained image shown in Figure 1B.

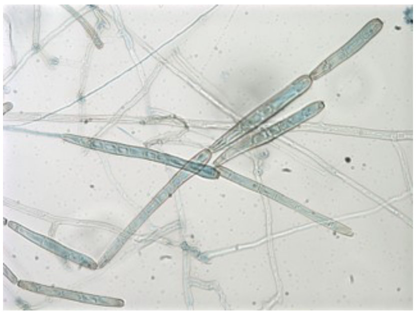

Figure S2: Morphological characteristics of *C. cassiicola* isolated from our index patient. Lactophenol cotton blue staining showing chaining of *C. cassiicola* conidia. Chains contained up to 9 conidia, which were 9-20 μm in length.

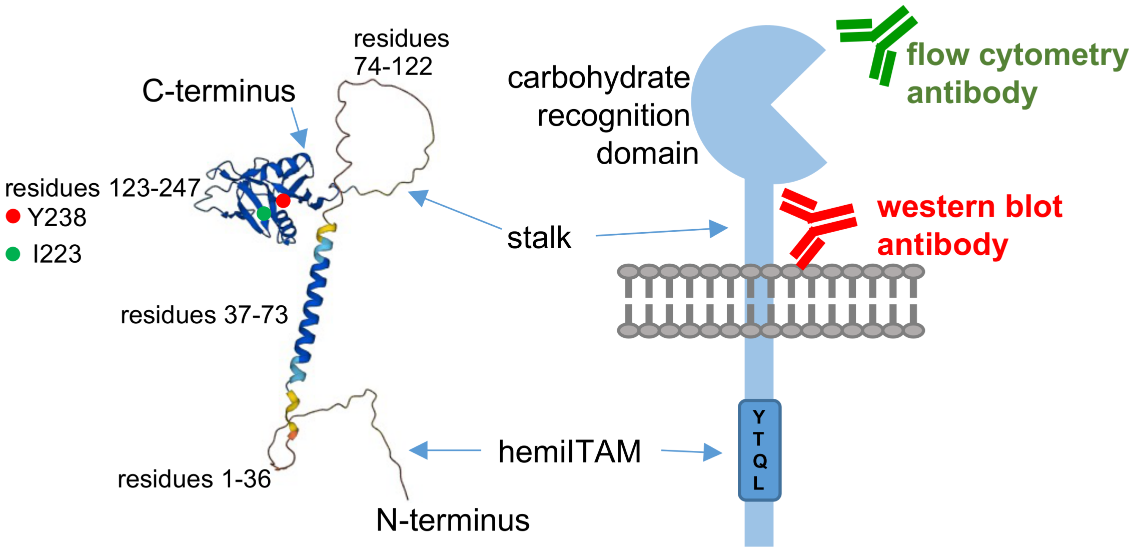

Figure S3: Human DECTIN-1 structure. Image on left is human DECTIN-1 structure as predicted by AlphaFold (blue are areas of high confidence, yellow is low confidence). Residues 1-36 are the intracellular tail containing the signalling ITAM motif, residues 37-73 are the transmembrane domain, residues 74-122 are the stalk domain, and residues 123-247 are the extracellular carbohydrate recognition domain (CRD). The two residues mutated in our index patient are highlighted with red (Y238) and green (I223) circles. The antibody used in Fig. 1D binds to the CRD, which is affected by the patient's mutations. The antibody used in Fig. 1E binds to the stalk region, which is not affected by the patient's mutations.

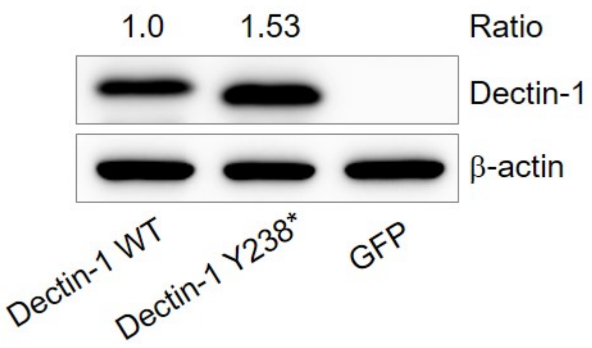

Figure S4: DECTIN-1 immunoblot analysis in transfected HEK293 cells. Representative protein immunoblot image of DECTIN-1 expression in HEK293 cells transfected with the WT or Y238* DECTIN-1 allele. β-actin was used as loading control.

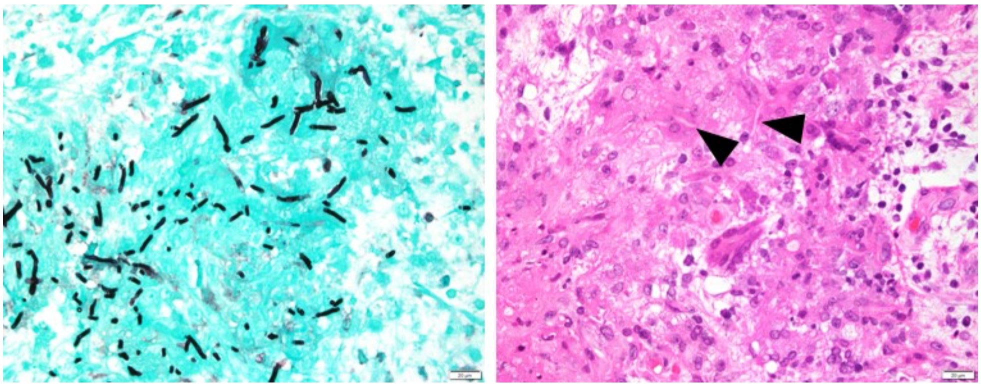

Figure S5: Ineffective granulomatous inflammatory response to *C. cassiicola* hyphae in a CARD9-deficient patient. Histology of biopsy sample taken from the brain lesion of a CARD9-deficient patient (CARD9.02) (imaged at 100X magnification), stained with GMS (left) and H&E (right). Both images are from consecutive cuts of the same biopsy sample. Black arrows highlight fungal cells visible in the H&E image. Scale bar 20μm.

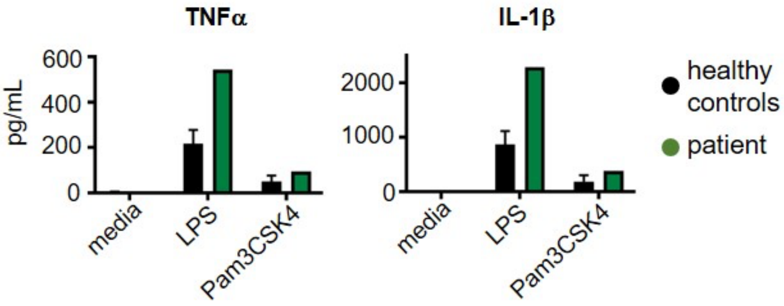

Figure S6: DECTIN-1 deficiency in our index patient does not impair cytokine responses to Toll-like receptor agonists. PBMC isolated from healthy donors (n=7) or our DECTIN-1-deficient index patient were stimulated with LPS or Pam3CSK4 *ex vivo* for 48 hours before measuring cytokine concentrations in the supernatant.

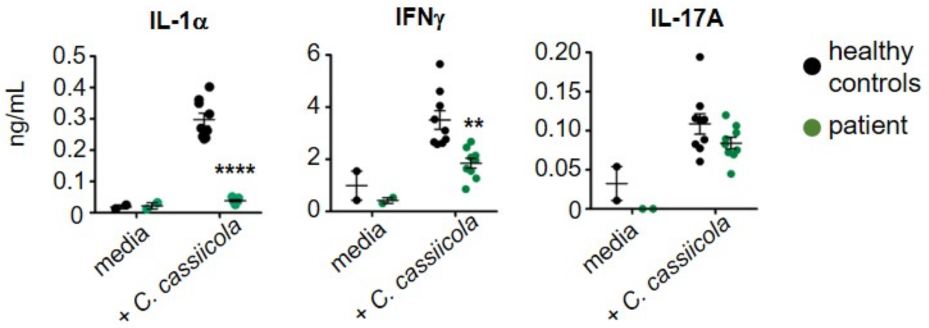

Figure S7: Production of IL-1α and IFN-γ but not IL-17A is impaired in DECTIN-1-deficient PBMC following *C. cassiicola* stimulation. PBMC isolated from healthy donors (n=2) or our DECTIN-1-deficient index patient (n=2 separate blood draws) were stimulated with *C. cassiicola ex vivo* for 48 hours before measuring cytokine concentrations in the supernatant. Each dot represents a technical replicate/well. Data was analyzed by two-way ANOVA with Bonferroni correction. ***P*<0.01, *****P*<0.0001.

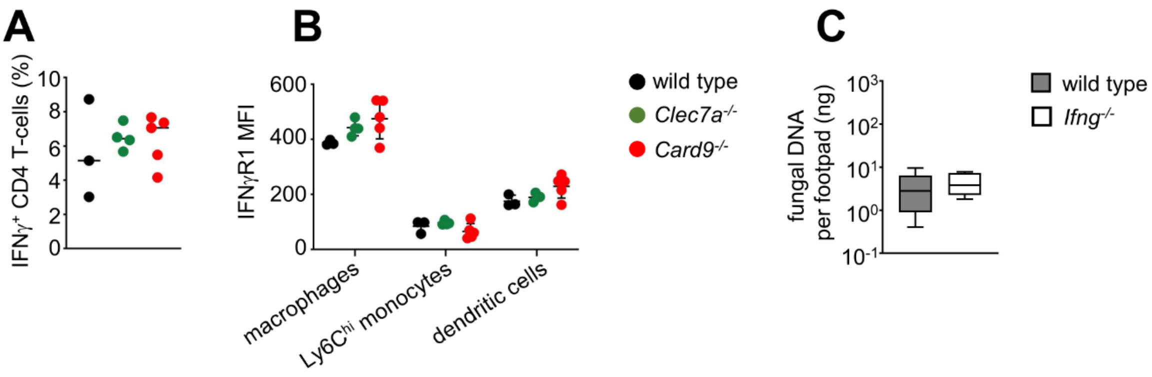

Figure S8: IFN-γ plays a redundant role in the protection against *C. cassiicola* subcutaneous infection in mice. Panel A shows the frequency of IFN-γ^+ $CD4^+$ T cells in the infected footpad at day 3 post-infection in WT (black dots, n=3), *Clec7a*$^{-/-}$ (green dots, n=4) and *Card9*$^{-/-}$ (red dots, n=5) mice**.** No measurable accumulation of other IFN-γ^+ innate and adaptive lymphoid cells was noted in the infected footpads of WT, *Clec7a*$^{-/-}$ or *Card9*$^{-/-}$ mice (not shown). Panel B shows mean fluorescent intensity (MFI) of IFNγR1 staining on the indicated myeloid cell populations in the *C. cassiicola*-infected footpad at day 3 post-infection. Panel C shows fungal burden (as measured by qPCR) in the footpad of WT (n=8) and IFNγ-deficient (n=8) mice at day 10 post-infection.

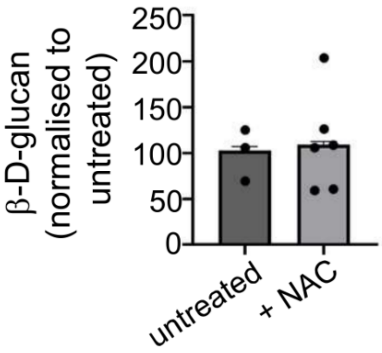

Figure S9: ROS is not required for *C. cassiicola* killing by macrophages. Bone marrow-derived mouse macrophages were cultured for 18 hours in the presence of 5 mM NAC to block ROS production or left untreated. Macrophages were challenged with *C. cassiicola* for 3 hours and β-D-glucan was measured in the supernatants. Data is pooled from 2 independent experiments. Bar graph shows the mean +/- SEM of the pooled data, the overlaid dot plot shows the technical replicates from one of these experiments.

Supplementary Tables

Table S1: Demographic information of healthy individuals used for determination of DECTIN-1-dependent cytokine production.

Control #	Age in years	Sex	Race
1	69	M	White
2	27	M	White
3	63	M	White
4	54	M	White
5	76	M	White
6	38	M	White
7	34	M	Asian
8	32	F	White

The 8 healthy individuals did not carry the deleterious CLEC7A mutations c.714T>G (p.Tyr238Ter) or c.668T>G (p.Ile223Ser) as determined by Sanger sequencing.

Table S2: Amplification primers and cycling conditions for *CLEC7A.*

primer	sequence 5' - 3'	anneal	extend (min)
x1aF	AAGAGGCAGTGTAGCGTAACAGAAAAGACCAC	56	02:20
x1aR	CAATAGGTTGCCCAAGAAAAGAATTACATCAG		
x2aF	CCACCGTGCAAGGCCAGATTTTTGATGAACTGTAG	59	01:00
x2aR	TGAGGGAGGCAGCAAGAGGCAAATGTCATAGAGTC		
x3aF	TTTTACCTTTCCAGGGTTATAAGCTAGTTAGTGGT	55	01:30
x3aR	AGCCAACTGCATAGGGTACTTATAATTTCACCTTA		
x4aF	CAATTTTTCATCTCTGGACCCATACCTTCTCACTA	56	01:30
x4aR	TTATGTTTTAACCCTCTTTGCCTTTCCCAGTATCT		
x5aF	CGCGCCCGGCCTTGCATTCTTTACC	60	01:00
x5aR	ACTGAGGCCTGGCTTCAAAACCCATGCTCTTA		
x6aF	GCCTTTAAATGGCCTGGGCATCT	55	01:00
x6aR	AGCTACTTGAGGGGCTGAGGCGAGAG		

Table S3: Sequencing primers for *CLEC7A.*

primer	sequence 5' - 3'
x1sF	TTAGTTGTGAGAGTAATGAAGGCAGCATAAG
x2sR	TTCAGCTCCGCTTGTTTTCTTCATTAATTTATCCT
x3sF	TGACATTTGCCTCTTGCTGCCTCCCTCATTC
x4sR	GCGGCTTCACTGCACTCTAGCTTGGGCAACAG
x5sR	ATGGCCTCACTCTTTTTCACTCTAAGGTCCTC
x6sF	TATCCTTACTAATTTATTTGTGCGCCATGTAGACA
x6sR	AATCACAGCCTCTCCCTTCAATTTCTTGGTT

Full unedited gel for Figure 1E

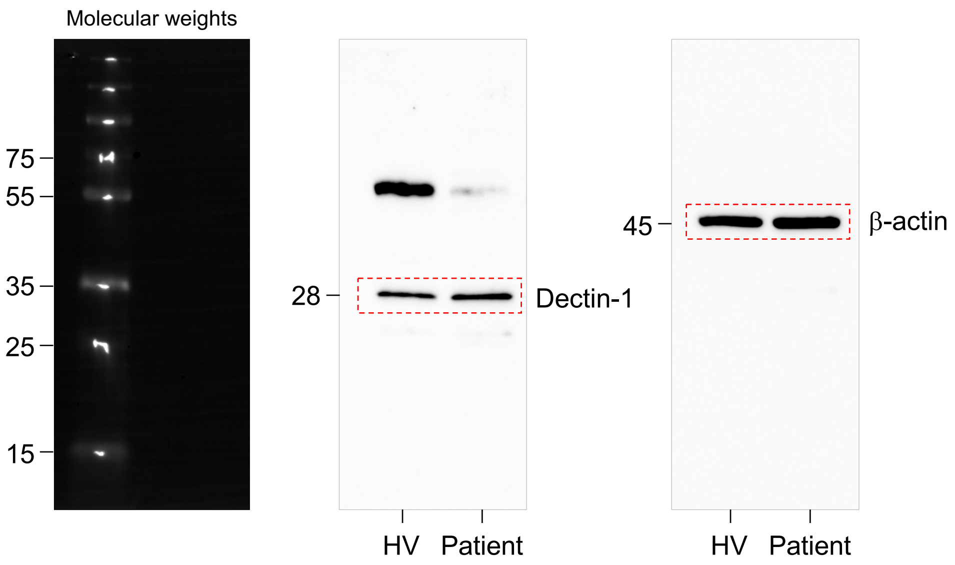

Full unedited gel for Figure S3

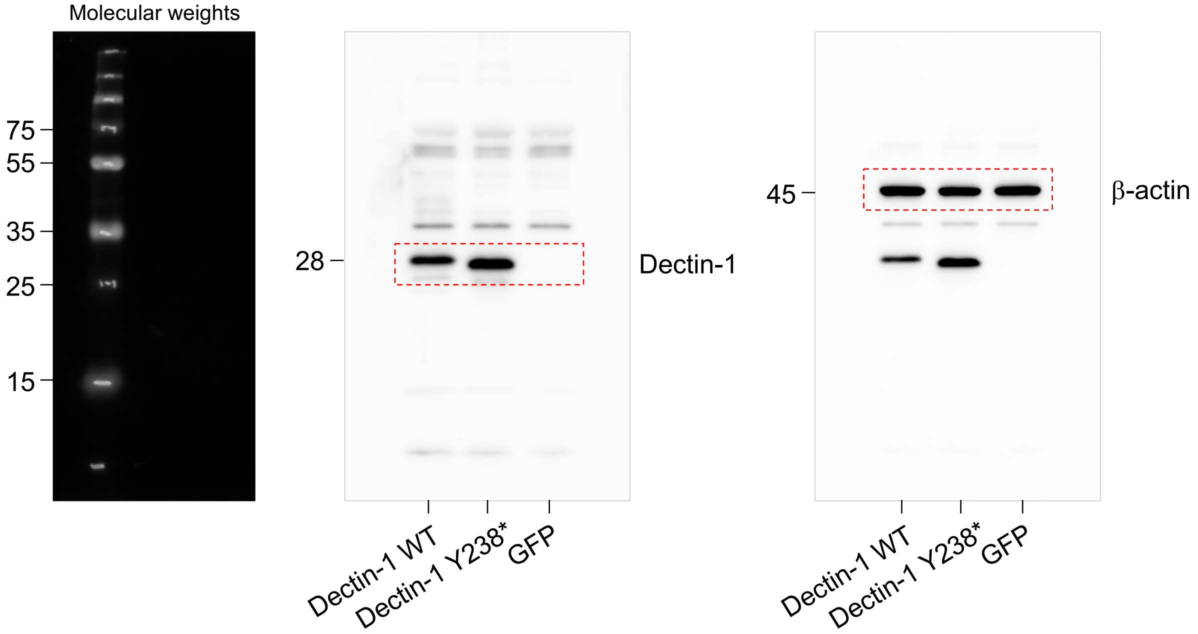

Supplement: Supplemental data [file jci-132-159348-s156.pdf]
